# Morphological and Stress Vulnerability Indices for Human Coronary Plaques and Their Correlations with Cap Thickness and Lipid Percent: An IVUS-Based Fluid-Structure Interaction Multi-patient Study

**DOI:** 10.1371/journal.pcbi.1004652

**Published:** 2015-12-09

**Authors:** Liang Wang, Jie Zheng, Akiko Maehara, Chun Yang, Kristen L. Billiar, Zheyang Wu, Richard Bach, David Muccigrosso, Gary S. Mintz, Dalin Tang

**Affiliations:** 1 Mathematical Sciences Department, Worcester Polytechnic Institute, Massachusetts, United States of America; 2 Mallinckrodt Institute of Radiology, Washington University, St. Louis, Missouri, United States of America; 3 The Cardiovascular Research Foundation, New York, New York, United States of America; 4 Network Technology Research Institute, China United Network Communications Co., Ltd., Beijing, China; 5 Biomedical Engineering Department, Worcester Polytechnic Institute, Worcester, Massachusetts, United States of America; 6 Cardiovascular Division, Washington University School of Medicine, St. Louis, Missouri, United States of America; 7 School of Biological Science and Medical Engineering, Southeast University, Nanjing, China; University of California San Diego, UNITED STATES

## Abstract

Plaque vulnerability, defined as the likelihood that a plaque would rupture, is difficult to quantify due to lack of in vivo plaque rupture data. Morphological and stress-based plaque vulnerability indices were introduced as alternatives to obtain quantitative vulnerability assessment. Correlations between these indices and key plaque features were investigated. In vivo intravascular ultrasound (IVUS) data were acquired from 14 patients and IVUS-based 3D fluid-structure interaction (FSI) coronary plaque models with cyclic bending were constructed to obtain plaque wall stress/strain and flow shear stress for analysis. For the 617 slices from the 14 patients, lipid percentage, min cap thickness, critical plaque wall stress (CPWS), strain (CPWSn) and flow shear stress (CFSS) were recorded, and cap index, lipid index and morphological index were assigned to each slice using methods consistent with American Heart Association (AHA) plaque classification schemes. A stress index was introduced based on CPWS. Linear Mixed-Effects (LME) models were used to analyze the correlations between the mechanical and morphological indices and key morphological factors associated with plaque rupture. Our results indicated that for all 617 slices, CPWS correlated with min cap thickness, cap index, morphological index with r = -0.6414, 0.7852, and 0.7411 respectively (p<0.0001). The correlation between CPWS and lipid percentage, lipid index were weaker (r = 0.2445, r = 0.2338, p<0.0001). Stress index correlated with cap index, lipid index, morphological index positively with r = 0.8185, 0.3067, and 0.7715, respectively, all with p<0.0001. For all 617 slices, the stress index has 66.77% agreement with morphological index. Morphological and stress indices may serve as quantitative plaque vulnerability assessment supported by their strong correlations with morphological features associated with plaque rupture. Differences between the two indices may lead to better plaque assessment schemes when both indices were jointly used with further validations from clinical studies.

## Author Summary

Cardiovascular diseases are closely related to atherosclerotic plaque progression and rupture. Early detection of vulnerable plaques and prediction of potential plaque rupture and related clinical events are of vital importance. Plaque vulnerability, defined as the likelihood that a plaque would rupture, is difficult to measure due to lack of in vivo plaque rupture data. Morphological and stress-based plaque vulnerability indices were introduced in this paper as alternatives to obtain quantitative vulnerability assessment with potential improvement of patient screening tools. In vivo intravascular ultrasound data were acquired from patients and computational coronary plaque models were constructed to obtain data for analysis and index assignments. For the 617 slices from the 14 patients, morphological and stress indices were assigned to each slice using methods consistent with American Heart Association plaque classification schemes. Correlation analyses were performed for all the morphological and mechanical factors considered. The stress index has 66.77% agreement with morphological index. Morphological and stress indices may serve as quantitative plaque vulnerability assessment supported by their strong correlations with morphological features associated with plaque rupture. Differences between the two indices may lead to better plaque assessment schemes when the complementary indices were jointly used with further validations from clinical studies.

## Introduction

Cardiovascular diseases (CVD), especially acute coronary syndromes, are closely associated with atherosclerotic plaque progression and rupture. Accurate assessment of plaque vulnerability is of ultimate importance to CVD research, diagnosis, prevention and proper treatment. Plaque vulnerability is defined as the likelihood that a plaque would rupture. While this vulnerability concept is well accepted, its quantification is extremely difficult due to lack of actual plaque rupture and clinical event data. In this paper, morphological plaque vulnerability index (morphological index) and stress-based plaque vulnerability index (stress index) will be introduced as plaque assessment using morphological and mechanical data. These indices provide computable quantitative plaque vulnerability measures for patient screening purpose using morphological and mechanical data while the absolute vulnerability is not available. Correlations between these indices and key plaque features including cap thickness and necrotic lipid-rich core (referred to as lipid thereafter) will also be investigated.

Atherosclerotic plaque progression and rupture are believed to be associated with morphological factors, plaque components, material properties, and mechanical stress/strain conditions, etc. [1–7]. More and more studies have shown that the mechanical forces play an important role in plaque progression and rupture process and should be considered with plaque morphology and compositions together for better plaque vulnerability assessment [1,8–12].

Considerable advances in medical imaging technology have made it possible to construct image-based computational models integrating plaque morphology, components, and mechanical stress/strain conditions and obtain plaque vulnerability assessment based on more complete information [12–13]. Based on in vivo magnetic resonance imaging (MRI) carotid plaque data with actual plaque rupture indicated by ulceration (n = 12, 5 with ulceration), Tang et al. reported that mean plaque wall stress (PWS) from all ulcer nodes in ruptured plaques was 86% higher than that from all non-ulcer nodes (123.0 vs. 66.3 kPa, p<0.0001). Mean flow shear stress (FSS) from all ulcer nodes in ruptured plaques was 170% higher than that from all non-ulcer nodes (38.9 vs. 14.4 dyn/cm^2^, p<0.0001) [12]. Mean critical PWS (CPWS) from the 5 ruptured plaques was 126% higher than that from the 7 non-ruptured ones (247.3 vs. 108 kPa, p = 0.0016 using log transformation) [12]. Using finite element analysis to calculate the plaque structural stress (PSS) for 4429 intravascular ultrasound (IVUS) frames from 53 patients, Teng et al. found PSS was higher in non-calcified thin-cap fibroatheroma and in patients with the factors contributing to the acute syndrome such as plaque burden > = 70%, mean lumen area < = 4mm^2^ [14]. Based on a comparative study of 40 patients with 20 symptomatic and 20 asymptomatic, Li et al. reported that lumen curvature and fibrous cap thickness were the major factors affecting plaque stress distribution. Furthermore lumen curvature in plaques of symptomatic patients was significantly larger compared to that of asymptomatic patients [15]. Based on the simulation results of endothelial shear stress (ESS) in the human coronary of 7 patients, Hetterich et al. claimed plaque prevalence was highest in areas of low ESS (49.6%) and followed by high ESS quartile (34.8%) while in parts exposed to intermediate-low and intermediate-high ESS quartiles, few plaques were found (20.0% and 24.0%) (p<0.001) [16]. In a comparison study between 54 asymptomatic and 45 acutely symptomatic patients, Zhu et al. reported that plaque wall stresses at critical vulnerable sites were significantly higher in acutely symptomatic group comparing to the asymptomatic group (median, inter quartile range: 198.0 kPa (119.8–359.0 kPa) vs 138.4 kPa (83.8–242.6 kPa), P = 0.04) [17]. In another multi-patient study based on in vivo MRI data of carotid plaques, Huang et al. demonstrated that mean PWS at the hemorrhage nodes were higher than that from the non-hemorrhage nodes (75.6 vs. 68.1 kPa), and mean FSS at hemorrhage nodes were also higher than that from the non-hemorrhage nodes (15.0 vs. 11.5dyn/cm^2^) [18]. A very noticeable finding for vulnerable plaque research was concerned with microcalcifications [19–23]. Vengrenyuk et al. [19–20], Bluestein et al. [21], Maldonado et al. [22], Cardoso and Weinbaum [2], and Kelly-Arnold et al. [23] demonstrated that plaque cap with micro-calcification inclusions are associated with elevated stress levels and may be related to plaque rupture.

For plaque progression studies, in a multi-patient IVUS-based follow-up study (n = 20), Samady et al. divided slices into low, intermediate, and high wall shear stress (WSS) groups and found that low-WSS segments demonstrated greater reduction in vessel (P<0.001) and lumen area (P<0.001), and high-WSS segments demonstrated an increase in vessel (P<0.001) and lumen (P<0.001) area [24]. In a follow-up study of 506 patients with acute coronary syndrome (ACS) to assess plaque natural history, Stone et al. reported that increase in plaque area was predicted by baseline large plaque burden; decrease in lumen area was independently predicted by baseline large plaque burden and low wall shear stress [25].

In this paper, in vivo intravascular ultrasound (IVUS) data were acquired to construct 3D models with fluid-structure interactions (FSI) and cyclic bending and obtain plaque morphological and mechanical stress/strain data for analysis. Six hundred and seventeen (617) slices were obtained from 14 patients. Based on morphological features available from IVUS Volcano Histology (VH), three morphological characteristic-related indices (lipid index, cap index, and morphological index) were introduced consistent with American Heart Association (AHA) plaque classifications [26]. Flow shear stress (FSS) and plaque wall stress/strain (PWS/PWSn) were collected at the critical sites in each slice to investigate their correlations with the morphological related factors. The Linear mixed-effect (LME) Models were used to analyze the correlation between each of the mechanical condition and each of the indices.

## Methods

### Data acquisition and modeling procedures

#### IVUS data and x-ray data

**Fig 1:**
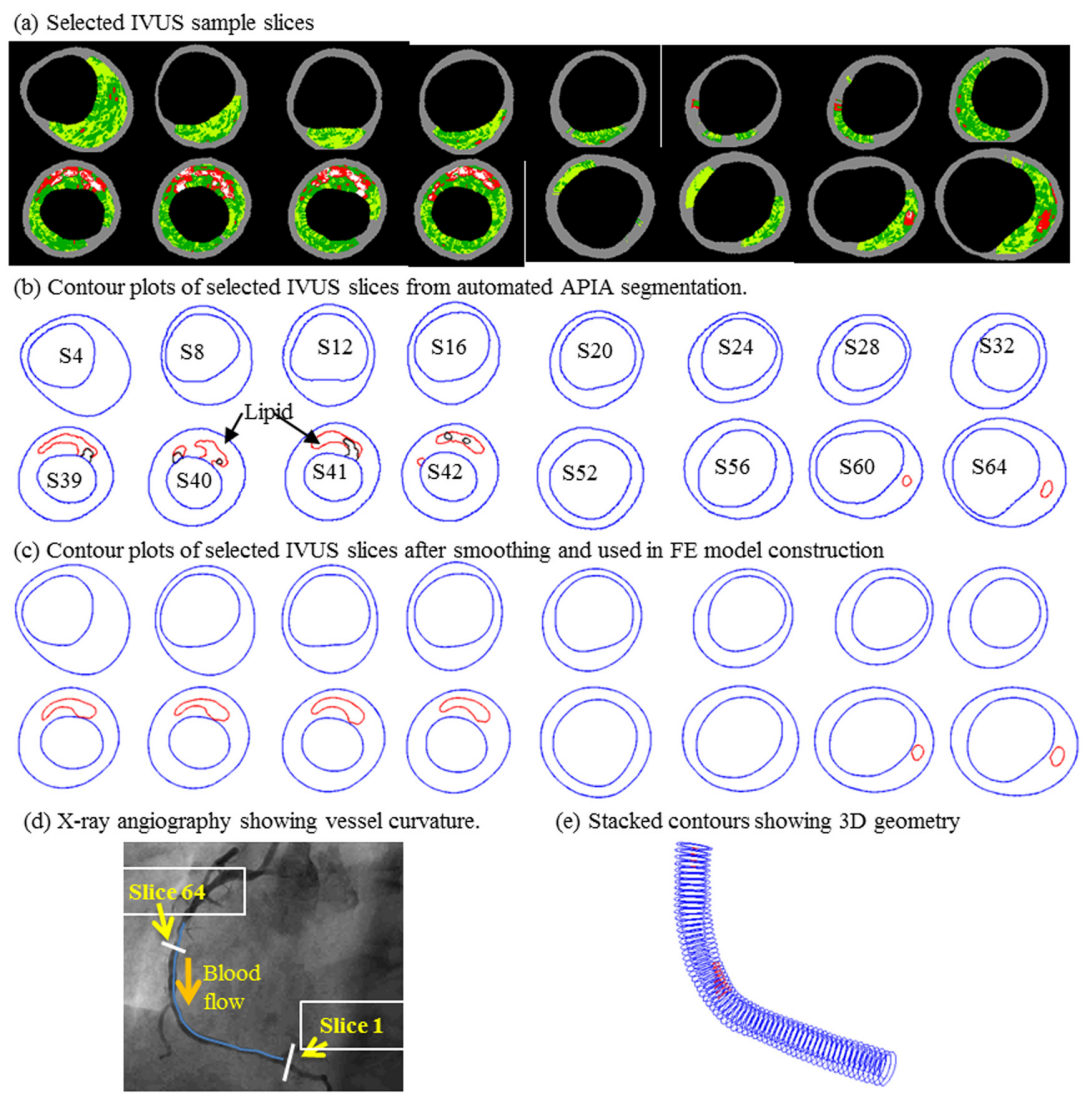
Samples of IVUS-VH figures and segmented contour plots, smoothed contour plots. Plots also include X-ray angiographic image and stacked segmented contours showing the 3D vessel geometry. Colors used in IVUS-VH: Red—necrotic core; White—dense calcium; Dark Green–Fibrous; Light Green—Fibro-Fatty.

The *in vivo* IVUS data of human coronary plaque were acquired from 14 patients (11M, 3F, Mean age: 59) at Cardiovascular Research Foundation (New York, NY) and Washington University at St. Louis after informed consent was obtained. This research has been approved by the Institutional Review Board (IRB) at Cardiovascular Research Foundation and the Institutional Review Board (IRB) at Washington University–St. Louis, respectively. A 20MHz, 2.9F phased-array transducer catheter (Eagle Eye Gold, Volcano Therapeutics, Rancho Cordova, CA) was used to acquire IVUS data. The original gray scale of IVUS image had an axial resolution of 150–200 μm and a lateral resolution of 150–300μm [27]. The IVUS-VH was created by using autoregressive models to provide 4 classified tissue components [28]. The principle of this technique is to combine information from both envelop amplitude of the reflected radiofrequency signals and underlying frequency content for analysis. Despite these relatively low resolution, the displayed spatial resolution or virtual resolution in the IVUS images were approximately 20μm, which was used to create contours of segmented plaque tissue. There were 617 IVUS slices data in total obtained from the 14 plaque models. The X-ray angiogram (AlluraXper FD10 System, Philips, Bothel, WA) was obtained and used to identify the location of the coronary artery stenosis, vessel curvature, and cyclic bending of the vessel due to the cardiac motion. Fig 1 shows selected sample IVUS-VH slices, segmented contours, contour after smoothing, X-ray angiography and the reconstructed 3D geometry of the vessel.

#### The FSI model

Our previous publications have demonstrated that fluid-structure interaction, cyclic bending, anisotropic material properties and the pre-shrink process were important for accurate stress/strain predictions [29–30] and those features were included in the models constructed in this paper. In our models, blood flow was assumed to be laminar, Newtonian, and incompressible. The Navier-Stokes equations with arbitrary Lagrangian-Eulerian (ALE) formulation were used as the governing equations. Pulsating pressure conditions were specified at the inlet and outlet using patient’s systole and diastole arm pressure conditions. Periodic displacement was prescribed to the inlet and outlet surfaces and the myocardium side of the vessel to simulate the cyclic bending movement obtained from X-Ray angiography. The vessel material was assumed to be hyperelastic, anisotropic, nearly-incompressible and homogeneous. A modified Mooney-Rivlin material model was used to describe the material properties of the human coronary. For simplicity, Plaque components (only calcification and lipid were segmented by IVUS-VH and included in this paper) assumed to be hyperelastic, isotropic, nearly-incompressible and homogeneous, and a simplified modified Mooney-Rivlin material model was used to describe the material properties of the plaque components. No-slip conditions and natural traction equilibrium conditions were assumed at all interfaces. More details can be found in Yang et al [30].

The strain energy density function of the modified Mooney-Rivlin material model was given below [31]:

$$\text{W}={\text{c}}_{1}({\text{I}}_{1}-3)+{\text{c}}_{2}({\text{I}}_{2}-3)+{\text{D}}_{1}[\exp({\text{D}}_{2}({\text{I}}_{1}-3))–1]+{\text{K}}_{1}/2{\text{K}}_{2}\{\exp[{\text{K}}_{2}{\left({\text{I}}_{4}\text{-}1\right)}^{2}\text{-}1]\}.$$ (1)

$${\text{I}}_{1}=\sum C_{ii},\;{\text{I}}_{2}=½[{\text{I}}_{1}{}^{2}\;\text{-}\;C_{ij}C_{ij}],$$ (2)

where I_1_ and I_2_ are the first and second invariants of right Cauchy-Green deformation tensor **C** defined as ***C*** = [*C*_*ij*_] = **X** ^T^ **X, X** = [X_ij_] = [∂x_i_/∂a_j_], (x_i_) is current position, (a_i_) is original position, *I*_4_ = *C*_*ij*_(**n**_*c*_)_*i*_(**n**_*c*_)_*j*_, **n**_*c*_ is the unit vector in the circumferential direction of the vessel, c_1_, c_2_,D_1_, D_2_, and K_1_ and K_2_ are material constants.

Biaxial testing was performed using eight coronary arteries from 4 cadavers (age: 50–81) and a two-step square-least method was conducted to fit our experimental data for the Mooney-Rivlin material model to get the material constants [32]. The parameters used in this paper were: c_1_ = -1312.9 kPa,c_2_ = 114.7 kPa, D1 = 629.7 kPa, D_2_ = 2.0, K_1_ = 35.9 kPa, K_2_ = 23.5. Our parameters are also consistent with data available in the literature [33–35].

#### Patient-specific plaque model construction and solution method

Patient-specific 3D plaque FSI models with FSI were constructed for all 14 patients and solved by ADINA (Adina R &D, Watertown, MA) following established procedures [29]. We divided the cardiac cycle to 50 steps so time step is roughly about 0.02 second (0.0175 s to 0.025 s). Three cycles were simulated (the third period was repeating the second period) which takes about 3–4 hours on our server. More details about our component-fitting mesh generation technique were described in Yang et al [30]. In particular, IVUS slices were stacked and orientations were checked manually to avoid sudden misalignments. In model construction process, small lipid cores and calcifications far away for lumen surface were deleted. 2-D smoothing was applied to plaque components with very choppy boundaries so that their shapes become more regular and easier for mesh generation. Component clusters (several components close to each other) were combined to a larger component using their envelop lines. 3D smoothing was applied so that the lumen surface, out-boundary, and plaque components would not have very sudden changes from one slice to next. Plaque 3D volume were preserved with each 2D slice lumen and out-boundary adjustment made following volume conservation law (2% error was allowed). Cap thickness was kept unchanged in the entire process. Mesh analysis was performed by refining mesh density by 10% until changes of solutions became less than 2%. Three cardiac cycles were simulated in our computational models and the solution in the third period was taken as the final result.

#### Data extraction

**Fig 2:**
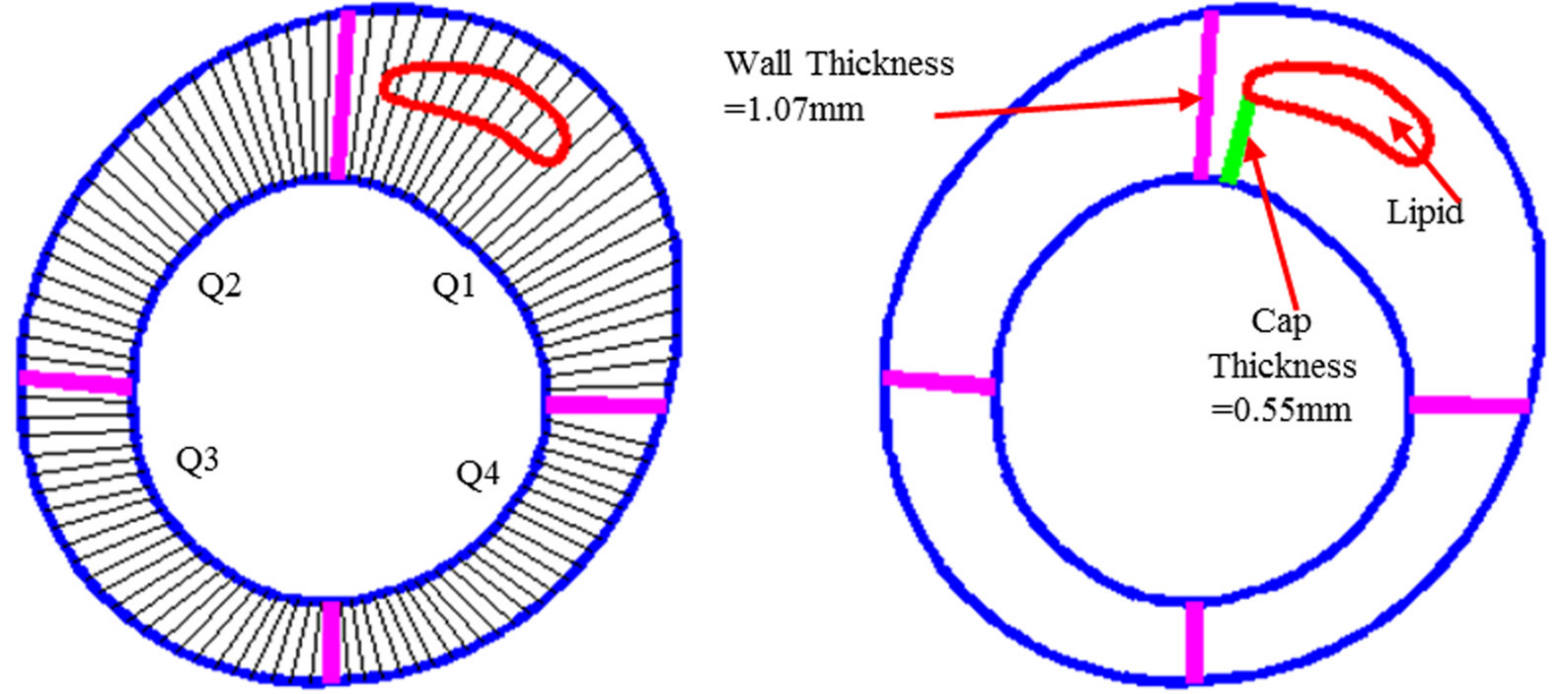
Sketch explaining definitions of quarters, wall thickness, cap thickness, and lipid (lipid-rich necrotic core).

For all the 617 IVUS slices, each slice was divided into 4 quarters with each quarter containing 25 evenly-spaced nodal points taken on the lumen, each lumen nodal point was connected to a corresponding point on vessel out-boundary. The length of the connecting line at a point is defined as the wall thickness at the nodal point. If the line passes a necrotic core or calcification block, the distance between the lumen point and the first time the line meets the necrotic core is defined as cap thickness. Fig 2 shows the 4 quarters of a sample slice with the connecting lines illustrating the definitions of wall thickness, cap thickness, and lipid depth.

The cap thickness (wall thickness, or lipid cap thickness, depends on whether this line passes a necrotic core or not, but calcification does not count in this case.) was calculated at each nodal point and the minimal values of cap thickness for all 100 points on each slice is selected to be the min capthickness. The local maxima of the mechanical factors (PWS, PWSn, FSS) from the thin cap (including the shoulder) were selected and recorded as critical PWS (CPWS), PWSn (CPWSn), FSS (CFSS) for this slice [11]. Lipid percentage for each slice was also calculated according to the formula:

Lipidpercentage=lipidarea/(outboundaryarea–lumenarea). (3)

#### Index assignments

Analogue to AHA classifications [8, 26] and based on current literature for morphological features related to plaque vulnerability [36–37], three indices with values from 0 to 4 were introduced to indicate the plaque vulnerability based on the morophological charactertics for each slice (see Table 1). Accroding to the lipid percentage of each slice, lipid index (with value 0, 1, 2, 3 or 4) was calculated to indicate the amount of lipid in the slice. The category with lipid index = 4 has the most amount of lipid while the category lipid index = 0 means there is little or no lipid in the slice. Similarly, each slice was categorized into four groups with cap index (0, 1, 2, 3 or 4) based on its min cap thickness. However, the category with cap index = 4 has the thinest cap thickness when plaque is more prone to rupture while the category cap index = 0 means the cap thickness is very thick, not easy for plaque to rupture. Furthermore, a morphological plaque vulnerability index (morphological index) was defined as the greater value of lipid index and cap index for a given slice, i.e.,

Morphological index=Max(cap index,lipid index). (4)

The criterion and threshold values for index value assignments based on morphological features are summarized in Table 1.

**Table 1:** The morphological features related indices classification: Cap index, Lipid index, Morphological index and comparison with AHA classifications [26, 36–37].

Cap index	Description	Lipid index	Description	Morpho-logical Index	AHA Types	Description	Vulnerability
0	No component	0	No Lipid	0	Type I or Type II	normal or slight intimal thickening	Very stable
1	Min Cap Thickness>0.2mm	1	Lipid Percentage <5%	1	Type III	moderate intimal thickening, no extracellular lipid, calcification or significant inflammation	Stable
2	0.15mm<Min CapThickness< = 0.2mm	2	5%< = Lipid Percentage <30%	2	Type IV, Vb, and Vc	small lipid core (<30% of plaque size); calcification may be present; thick fibrous cap (> 150 μm);	Slightly Unstable
3	0.065mm<Min Cap Thickness< = 0.15mm	3	30%< = Lipid Percentage <40%	3	Type Va	moderate lipid core (30–40% of plaque size) and fibrous cap (65–200μm); moderate intraplaque hemorrhage and inflammation	Moderately Unstable
4	Min Cap Thickness< = 0.065mm	4	Lipid Percentage > = 40%	4	Type VI.	large lipid core(>40%); thin fibrous cap(<65 μm); large intraplaque hemorrhage; extensive inflammation; previous plaque rupture	Highly Unstable

To use plaque stress for plaque vulnerability assessment, each slice was also assigned a stress plaque vulnerability index (stress index, with value 0, 1, 2, 3 or 4) according to its CPWS value. Stress index values were given by using five stress intervals which were determined to have the best match rate with morphological index. The stress intervals with the best agreement rates are given by Table 2. Correlations between CPWS and lipid percentage, min cap thickness, and those three morphological indices was analyzed using the Linear Mixed-Effects (LME) model [8] and the agreement between stress index and those three morphological indices was also performed.

**Table 2:** The stress interval to assign the stress index and the distribution of the stress index.

Stress index	0	1	2	3	4
Stress interval (unit kPa)	(0,35]	(35,55]	(55,89]	(89,140]	(140,∞)
Number of slices	16	101	211	189	100

## Results

**Fig 3:**
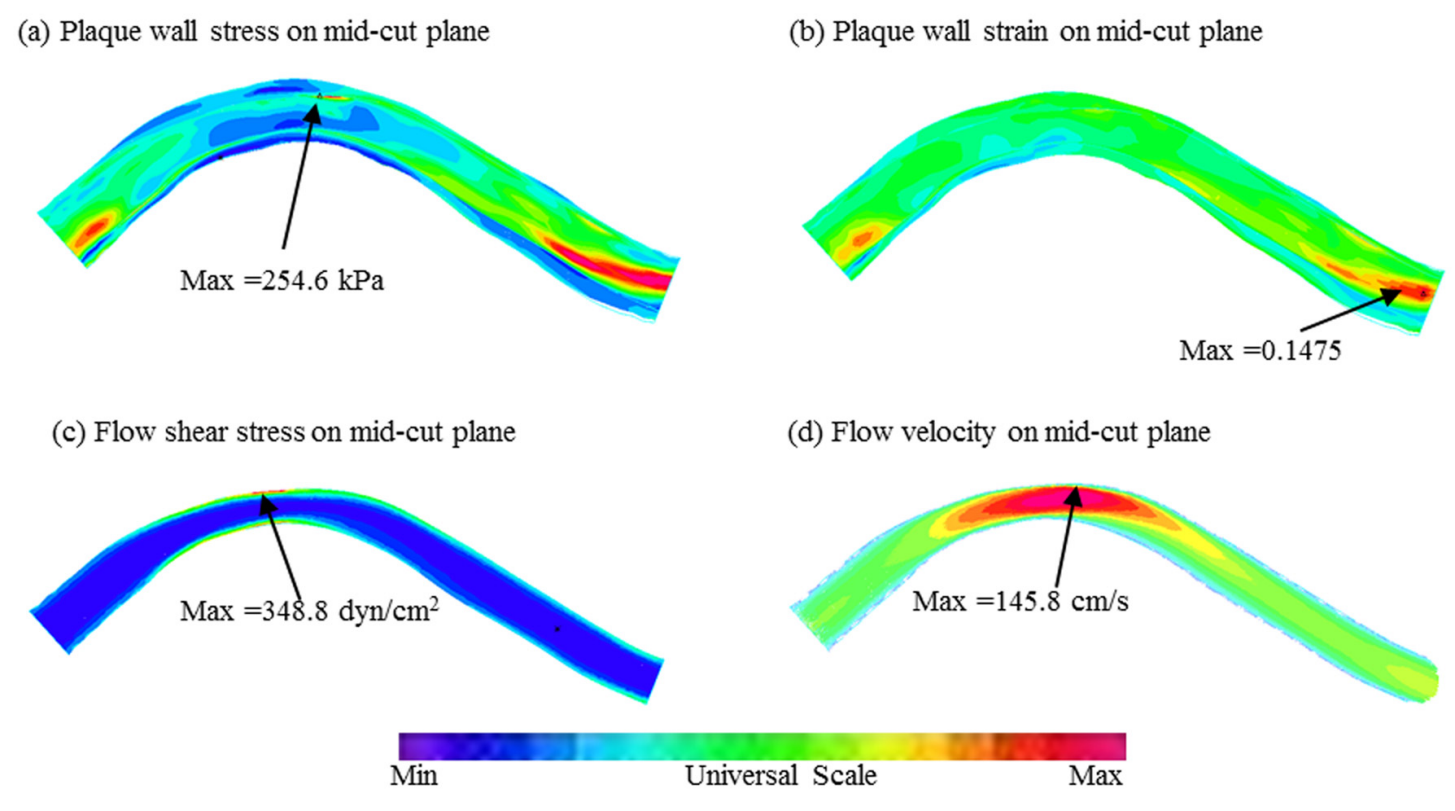
Samples plots of plaque wall stress, strain, wall shear stress and velocity on a mid-cut plane showing basic solution features.

Fig 3 gives plots of PWS, PWSn, FSS and flow velocity of one plaque at a mid-cut plane showing basic solution features from our FSI models. Using min cap thickness, lipid percentage, and mechanical stress/strain data obtained from our FSI models, correlation analysis between mechanical conditions and morphological factors/indices were performed for all 617 slices as a whole dataset. Correlation results for each patient were also presented.

### CPWS correlates significantly with morphological index, cap thickness and lipid percentage

**Fig 4:**
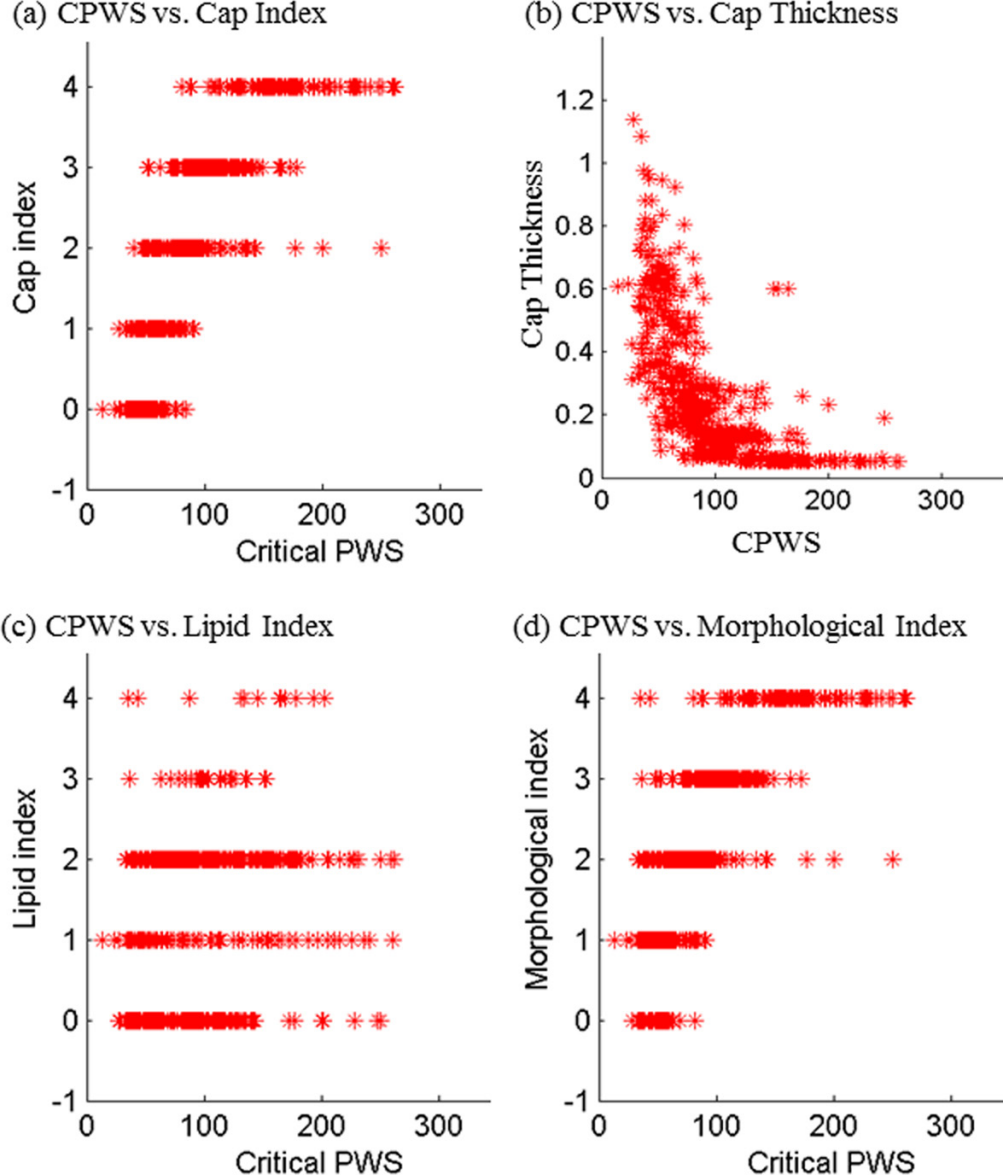
Scatter plots of CPWS vs cap index, min cap thickness, lipid index, and morphological index.

Table 3 summarized the correlation results between the three mechanical factors (CPWS, CPWSn, and CFSS) and 5 morphological factors: min cap thickness, cap index, lipid percentage, lipid index and morphological index. CPWS correlated significantly with each of the five morphological factors. There was a significant correlation between CPWS and the morphological index with correlation coefficient r = 0.7411, p-value<0.0001. Cap index (r = 0.7852) showed a slightly stronger correlation with CPWS than min cap thickness (r = -0.6414). The correlations showed opposite sign because thinner cap thickness had higher cap index. Lipid percentage and lipid index yielded weaker correlation than CPWS with correlation coefficient r = 0.2445 and r = 0.2338, respectively. Fig 4 shows the distribution of the CPWS with respect to cap index, min cap thickness, lipid index and morphological index.

**Table 3:** Correlation results between mechanical factors (CPWS/CPWSn, CFSS) and five morphological related factors (min cap thickness, cap index, lipid percentage, lipid index, and morphological index).

CPWS v.s.	Min cap thickness	Cap index	Lipid percent	Lipid index	Morphological index
r	-0.6414	0.7852	0.2445	0.2338	0.7411
p-value	<0.0001	<0.0001	<0.0001	<0.0001	<0.0001
CPWSn v.s.	Min cap thickness	Cap index	Lipid percent	Lipid index	Morphological index
r	-0.23067	0.2224	-0.1686	-0.2008	0.1679
p-value	<0.0001	<0.0001	0.0149	0.0002	<0.0001
CFSS v.s.	Min cap thickness	Cap index	Lipid percent	Lipid index	Morphological index
r	0.0239	-0.0419	0.1144	0.0825	-0.0357
p-value	***0*.*4126***	***0*.*1016***	0.0109	0.0101	***0*.*1430***

### CPWSn and CFSS had weaker or no correlations with morphological index, min cap thickness and lipid percentage

Statistically significant correlations were also observed between the CPWSn and 5 morphological factors (min cap thickness, cap index, lipid percentage, lipid index and morphological index) based on results from the 617 slices (see Table 3). Correlation results between CPWSn and min cap thickness, cap index and morphological index are similar to those of CPWS, but much weaker. CPWSn had negative correlation with lipid percentage and lipid index which is different from the results of CPWS. CFSS had significant correlations with lipid percentage and lipid index with r = 0.1144 and r = 0.0825, respectively. However, CFSS did not have significant correlations with min cap thickness, cap index, and morphological index.

It is natural that min cap thickness and CPWS correlate strongly. However, lipid content does not have similar strong correlation because some lipid core could have very thick cap. CFSS and min cap thickness did not have significant correlation because they do not correspond very well.

### Individual patient correlation analysis between CPWS and min cap thickness, cap index, lipid percentage, lipid index, morphological index

Individual patient correlations between CPWS and min cap thickness, cap index, lipid percentage, lipid index, morphological index were obtained for each of the 14 plaques and results are summarized in Table 4. For all 14 plaques, CPWS had significant positive correlations with cap index and morphological index, and negative correlation with min cap thickness. On the other hand, correlations between CPWS and lipid percentage, lipid index showed mixed results for the 14 patients. The number of patients showing positive/no significant/negative correlation between CPWS and lipid percentage were 4/10/0 respectively, while only 5 out of 14 patients showed significant positive correlation between CPWS and lipid index and the remaining 9 showed no correlation.

**Table 4:** Correlation results between five morphological features (min cap thickness, cap index, lipid percentage, lipid index, and morphological index) and CPWS for each patient on patient level (p-values for no significance results used bold italic).

Patient #	Slice number	CPWS v.s.	Min cap thickness	Cap index	Lipid percentage	Lipid index	Morphological index
P1	44	r	-0.6024	0.8311	0.0199	-0.0142	0.7272
		P-value	0.0004	0.0000	***0*.*9151***	***0*.*9259***	0.0000
P2	44	r	-0.7965	0.9132	0.7404	0.7545	0.8758
		P-value	0.0000	0.0000	0.0000	0.0000	0.0000
P3	34	r	-0.6624	0.8858	0.2043	0.4836	0.8858
		P-value	0.0001	0.0000	***0*.*3216***	0.0206	0.0000
P4	45	r	-0.7291	0.8351	-0.1032	0.0044	0.8351
		P-value	0.0000	0.0000	***0*.*6815***	***0*.*9831***	0.0000
P5	45	r	-0.6628	0.7900	0.1196	-0.0898	0.7441
		P-value	0.0000	0.0000	***0*.*5192***	***0*.*5882***	0.0000
P6	41	r	-0.7500	0.8745	0.3988	0.5072	0.8908
		P-value	0.0000	0.0000	0.0327	0.0027	0.0000
P7	57	r	-0.7973	0.8003	0.0241	-0.0230	0.6710
		P-value	0.0000	0.0000	***0*.*8754***	***0*.*8772***	0.0000
P8	57	r	-0.4071	0.4008	-0.2458	-0.1905	0.3849
		P-value	0.0000	0.0000	***0*.*0628***	***0*.*1835***	0.0000
P9	34	r	-0.4926	0.7154	-0.0881	0.0381	0.7154
		P-value	0.0062	0.0000	***0*.*6661***	***0*.*8106***	0.0000
P10	34	r	-0.7737	0.9067	0.6741	0.4714	0.9360
		P-value	0.0000	0.0000	0.0004	0.0092	0.0000
P11	36	r	-0.6249	0.6815	-0.2101	0.0457	0.7097
		P-value	0.0007	0.0000	***0*.*2171***	***0*.*7886***	0.0000
P12	36	r	-0.8477	0.8487	0.3086	0.2496	0.8478
		P-value	0.0000	0.0000	0.0351	***0*.*0866***	0.0000
P13	55	r	-0.6624	0.7459	0.0168	0.1870	0.6992
		P-value	0.0000	0.0000	***0*.*9316***	***0*.*2394***	0.0000
P14	55	r	-0.5897	0.8392	0.1983	0.2521	0.5951
		P-value	0.0000	0.0000	***0*.*0998***	0.0324	0.0000

### Correlation between stress index and min cap thickness, cap index, lipid percentage, lipid index, morphological index

Table 5 shows that correlation results of the stress index is similar to those of CPWS. There are significant correlations between stress index and all 5 morphological indices and factors. The correlation coefficient between stress index and cap index is the highest with r = 0.8185. There is a strong correlation between stress index and morphological index with r = 0.7715. The correlation coefficients between stress index and min cap thickness, lipid percentage and lipid index were r = -0.7127, 0.3139, 0.3067 respectively, all with p< 0.0001.

**Table 5:** Correlation results between stress index and five morphological related factors (min cap thickness, cap index, lipid percentage, lipid index, morphological index).

Stress index v.s.	Min cap thickness	Cap index	Lipid percentage	Lipid index	Morphological index
r	-0.7127	0.8185	0.3139	0.3067	0.7715
p-value	0.0000	0.0000	0.0000	0.0000	0.0000

### Agreement rates between stress index and cap index, lipid index and morphological index

For the 617 slices, each slice was assigned a stress index and 3 morphological related indices: cap index, lipid index and morphological index. Table 6 gives the agreement rates between stress index and cap index, lipid index, and morphological index. The matching rates (two indices have the same value for a slice) between stress index and cap index, lipid index and morphological index were 57.37%, 25.93% and 66.77% respectively. The result demonstrated the stress index has a strong agreement with morphological feature closely related to the plaque vulnerability, and could be used to aid the assessment of plaque vulnerability and plaque rupture.

**Table 6:** Agreement rates between stress index and cap index, lipid index and morphological index.

Stress index v.s	Cap index	Lipid index	Morphological index
Matched #	354	160	412
unmatched #	263	457	205
Matched rate	0.5737	0.2593	0.6677

## Discussion

### Significance of morphological and stress-based plaque vulnerability indices

Quantification of plaque vulnerability by its strict definition based on in vivo data is not currently realistic due to its statistical nature and lack of available in vivo data for plaque rupture and rupture-related clinical events. The morphological and stress-based plaque vulnerability indices introduced in this paper provided a possible approach for quantitative plaque vulnerability assessment using features known to be closely associated with plaque rupture. These indices may get closer to serve as appropriate approximations to plaque vulnerability as more plaque rupture and clinical event data are investigated and continued research adds more supporting evidence for the use of those alternative indices.

### Different indices may complement each other for better assessment

Currently, histology is serving as the gold standard for plaque vulnerability assessment. It has been well established that cap thickness and lipid size are closely related to plaque vulnerability. Our stress index was introduced trying to have best match with the morphological index. The stress index had a 66.77% agreement rate with morphological index. It should be emphasized that the disagreement between stress index and morphological index may lead to better plaque assessment strategies when both indices were used together to supplement each other. Large lipid core gives a higher morphological index. However, if its cap is thick, the slice may have a lower stress index. Large curvature causes higher CPWS which leads to a high stress index, which the morphological index may be low due to cap thickness and lipid size features. Further investigations are needed to investigate the differences of the two indices and seek for potential improvement on plaque assessment techniques and patient screening tools.

### Comparison of CPWS and CFSS correlation results

It is clear that CPWS and min cap thickness have strong correlations. However, CFSS did not have significant correlations with min cap thickness and cap index. CFSS showed weak positive correlation with lipid percentage and lipid index. This may be related the fact the higher CFSS values are associated to lumen narrowing, thicker vessel wall and larger lipid core. In general, CFSS is only one value for a slice and FSS from entire vessel should be used for further investigations [8].

### Potential improvement on quantification of plaque features and vulnerability indices

Several factors such as cap thickness and lumen surface inflammation are known to be related to plaque vulnerability and quantitative measurements of those features will bring improvement to our index accuracies. Among several modalities, Optical Coherence Tomography (OCT) may help advance our ability to diagnose vulnerable from non-vulnerable plaques to enhance future risk prediction and effective treatment for patients. OCT uses light rather than ultrasound; has a resolution of 15–20 microns and therefore can measure fibrous cap thickness; can classify plaque as fatty, fibrotic, calcific, or thrombotic; and can detect thin-cap fibroatheromas and plaque erosions in patients presenting with an acute coronary syndrome. The major limitations of OCT are penetration so that overall plaque burden cannot be measured and the inability to image through blood or penetrate red thrombus. We are currently working on techniques combining IVUS with OCT to obtain good overall plaque 3D geometry with better cap thickness quantifications.

### Limitations

Some other limitations of this study include: a) Patient-specific and tissue-specific material properties were not available for our study. We are currently investigate other approaches to measure patient-specific material properties in vivo; b) Currently, IVUS resolution is at 100–150 microns, mostly depending on the transducer frequency, which is not enough to clearly identify the thin caps with thickness under 100 micron. Optical coherence tomography can be used jointly with IVUS procedures and can provide spatial resolution of 10–20 microns. These combined approaches are also under investigation; c) While patient-specific angiography was used to extract vessel curvature variations in cardiac cycle for our cyclic bending, only angiography from one view angle was used. 3D curvature re-constructed from multiple angiography images with different (preferably orthogonal) view angles should be used in our future models [24]; d) Because IVUS data does not contain the adventitial layer of the vessel, our model also did not include that, as this is done in the current literature. It is arguable if simply adding a layer without actual data support would give us a better model. The adventitial layer should have considerable effect on stress/strain calculations. So our results should be understood with that simplification; e) Microcalcifications were not included in the current morphological index due to limitations of imaging; f) Interaction between the heart and vessel were not considered in this model. A model coupling heart motion and coronary bending would be designed when required data become available.
